# Swimming dynamics of screw-shaped untethered magnetic robots in confined spaces

**DOI:** 10.1007/s11071-025-11646-7

**Published:** 2025-08-11

**Authors:** Luuc de Jongh, Anke Klingner, Leendert-Jan W. Ligtenberg, Marcus C. J. de Boer, Jaap van der Kooij, Roger Lomme, Dorothee Wasserberg, H. Remco Liefers, Pascal Jonkheijm, Michiel C. Warlé, Islam S. M. Khalil

**Affiliations:** 1 RAM—Robotics and Mechatronics, University of Twente, 7500 AE Enschede, The Netherlands; 2 Department of Physics, German University in Cairo, El-Tagamoa El-Khames, New Cairo, Cairo 11835 Egypt; 3 Radboud University Medical Center, 6525 GA Nijmegen, The Netherlands; 4 LipoCoat B.V., 7521 AE Enschede, The Netherlands; 5 Laboratory of Biointerface Chemistry, TechMed Centre, University of Twente, 7500 AE Enschede, The Netherlands; 6 Technical Medical Centre, University of Twente, 7500 AE Enschede, The Netherlands

**Keywords:** Untethered magnetic robots, Confinement, Swimming dynamics, Wireless vascular navigation, *ex vivo*

## Abstract

In confined environments, the propulsion speed of torque-driven helical untethered magnetic robots (UMRs) depends on vessel diameter and helical pitch. This phenomenon likely affects the swimming speed of these robots when navigating *in vivo* within blood vessels. Achieving a consistent swimming speed across a range of vessel diameters is therefore essential for precise control of UMRs. With this goal, we investigate how vessel diameter and helical pitch influence UMR propulsion to identify optimal robot designs. UMRs from two groups–fixed length (FL) and fixed wave (FW)–were analyzed. In the FW group, longer robots achieved higher speeds in larger vessels due to better vessel guidance, whereas shorter robots performed best in medium-sized vessels. The FL group showed peak speeds at intermediate normalized wavenumbers ($\nu \approx 1$ to 1.8), significantly influenced by robot geometry and vessel confinement. Normalized wavenumber affected swimming speed and stability, with maximum efficiency at $\nu \approx 1$ and stability increasing with higher wavenumbers. Swimming performance was modeled using Stokeslets method and additional effects were considered by correction factors to improve agreement of experimental and modeling results. *Ex vivo* tests under physiological conditions demonstrated clear performance differences between robust (FL-9 ($\nu =2.3$), FW-5 ($\nu =2$)) and unstable swimmers, with robust designs showing consistent correlation between phantom and ex vivo trials. Unstable designs exhibited unpredictable behavior, emphasizing the importance of specific design parameters for stability and maneuverability. These findings highlight critical design factors–such as the normalized wavenumber, body length, and degree of vessel confinement–that are essential for enhancing UMR propulsion efficiency and control consistency, which are fundamental for successful navigation in targeted vascular biomedical applications.

**Supplementary Information:**

The online version contains supplementary material available at 10.1007/s11071-025-11646-7.

## Introduction

Untethered magnetic robots (UMRs), typically ranging from micrometers to millimeters in size, represent a promising class of microrobotic systems for navigating confined environments such as blood vessels. These robots are envisioned to perform a wide range of medical tasks, including targeted therapy delivery, minimally invasive surgery, and precise disease diagnosis [1–9]. For example, future detection of cancer cells could be facilitated by non-linear resonance interactions between UMRs and vessel walls [10]. Despite their potential, UMRs face significant engineering and biological challenges. These include reliable power supply, precise navigation control in complex physiological environments, biocompatibility, and the seamless integration of sensing and therapeutic functionalities. UMRs must also be capable of maneuvering through blood vessels to perform tasks such as cutting, grasping, transporting tissue samples, or delivering drugs to specific targets. Locomotion is further complicated by physiological factors such as pulsatile blood flow, tissue viscoelasticity, and forces arising from adhesion and surface tension. Moreover, blood viscosity, which directly affects UMR propulsion, varies with shear rate and shows substantial person-to-person variability, with an apparent viscosity of approximately 3.5 Cp. Large vessels such as the aorta, medium-sized arteries, vena cava, and veins have diameters in the range of 4 to 30 mm, flow velocities between 3 and 1000 mm/s, and Reynolds numbers ($\text {Re}$) greater than 150 [11]. At low Reynolds numbers and close proximities to vessel walls ($s<20\mathcal {L}/\textrm{Re}$, where $\mathcal {L}$ is a characteristic length), robot-wall interactions become significant and cannot be neglected [12].

The locomotion of rotating helical structures–inclu-ding rods, ribbons, and screws–in both unconfined and confined fluidic environments has been widely investigated through asymptotic methods and computational fluid dynamics simulations [9, 13] , numerical methods [14] or method of Stokeslets [15]. These studies demonstrate that geometric parameters, fluid properties, and wall interactions strongly influence torque, rotation rate, propulsion efficiency, and overall mobility. Typically, the propulsion speed increases with structural amplitude and eccentric positioning. There exists an optimal pitch-to-wavelength ratio that maximizes swimming velocity, and prior studies have shown that helical swimmers often benefit from confinement [14, 16–21]. Analogous to engineered systems [22–24], biological organisms such as sperm cells and nematodes effectively exploit flagellar or helical oscillatory and rotational motions to swim efficiently through confined anatomical spaces [25–28].

**Fig. 1:**
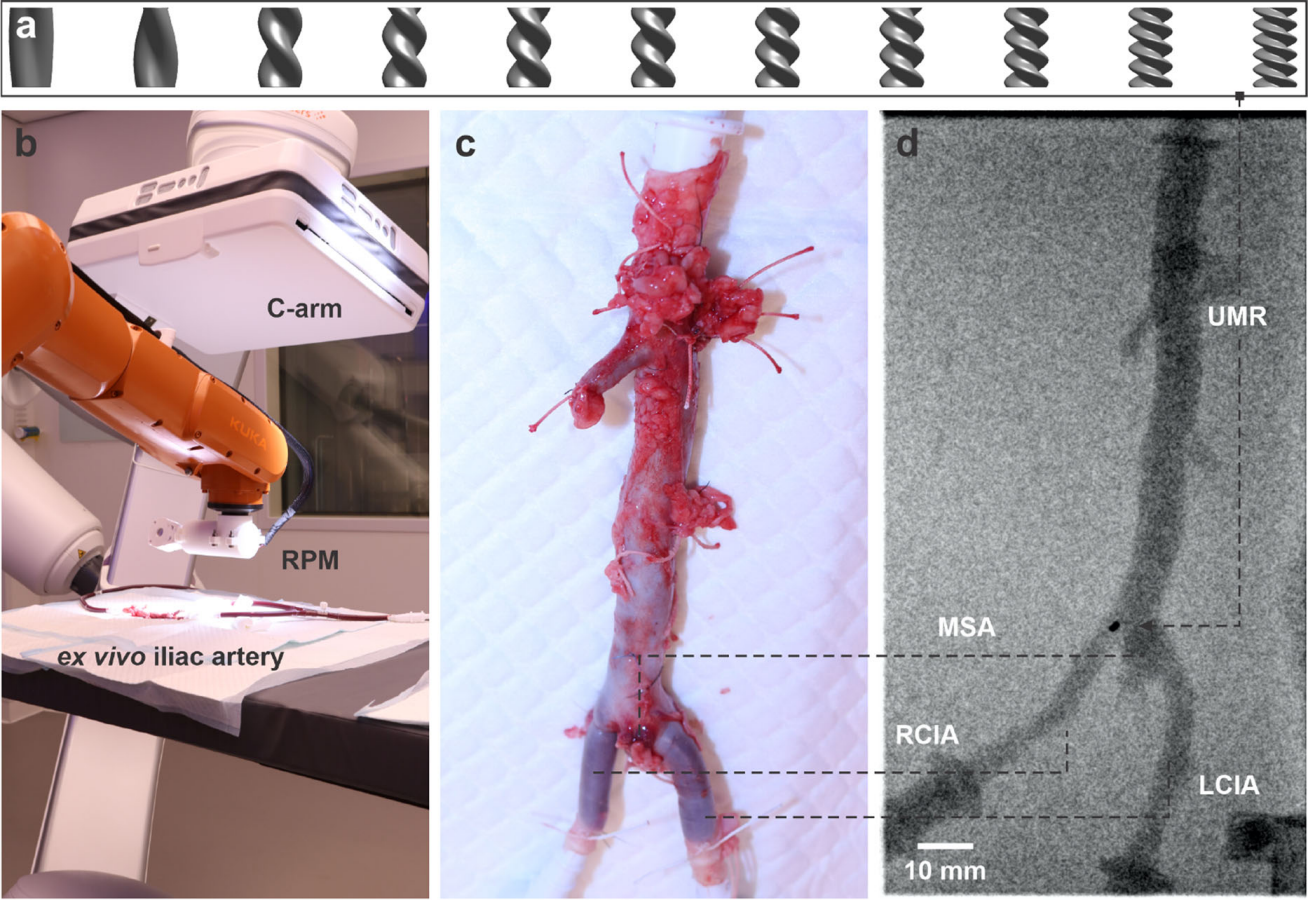
Overview of the *ex vivo* setup for testing differently designed untethered magnetic robots (UMRs). a) A set of UMRs from the fixed-length (FL) group, each with a length of 7.5 mm and radius of 2.1 mm, but varying normalized wavenumbers ranging from 0.25 (left) to 3.5 (right). b) The UMRs are actuated via externally applied rotating magnetic fields using a robotic arm and are monitored under C-arm fluoroscopy within a clinical environment. c) Optical image showing the anatomical layout of the vessels, with the large-diameter aorta at the top and the smaller-diameter iliac artery at the bottom. d) The X-ray image highlights the position of the UMR within the vessel, where the two black dots indicate the embedded permanent magnets. The image shows key anatomical structures, including the left common iliac artery (LCIA), the middle sacral artery (MSA), and the right common iliac artery (RCIA)

To emulate this natural efficiency, UMRs can be fabricated with biologically inspired helical shapes (Fig. 1a) and actuated using external magnetic fields (Fig. 1b) [1, 14, 23, 29–34]. Strong magnetic fields and gradients, such as those used in medical magnetic resonance imaging systems, have been employed to manipulate soft-magnetic beads *ex vivo* and *in vivo* in living swine [35–37]. However, precise control of UMRs remains limited due to unpredictable variations in propulsion speed and direction under confinement–resulting from complex interactions between geometry, hydrodynamics, and wall effects. These variations become especially pronounced during transitions between vessels of differing diameters, such as when the UMR moves from the larger abdominal aorta into the narrower left or right common iliac arteries (LCIA or RCIA), as shown in Figs. 1c and d. In such regions, the change in vessel diameter, combined with the helical pitch-to-wavelength ratio of the robot, significantly influences propulsion dynamics. These location-dependent effects encountered during vascular navigation highlight the need to improve the predictability and consistency of UMR swimming behavior across diverse anatomical geometries–an essential requirement for reliable clinical translation.

**Fig. 2:**
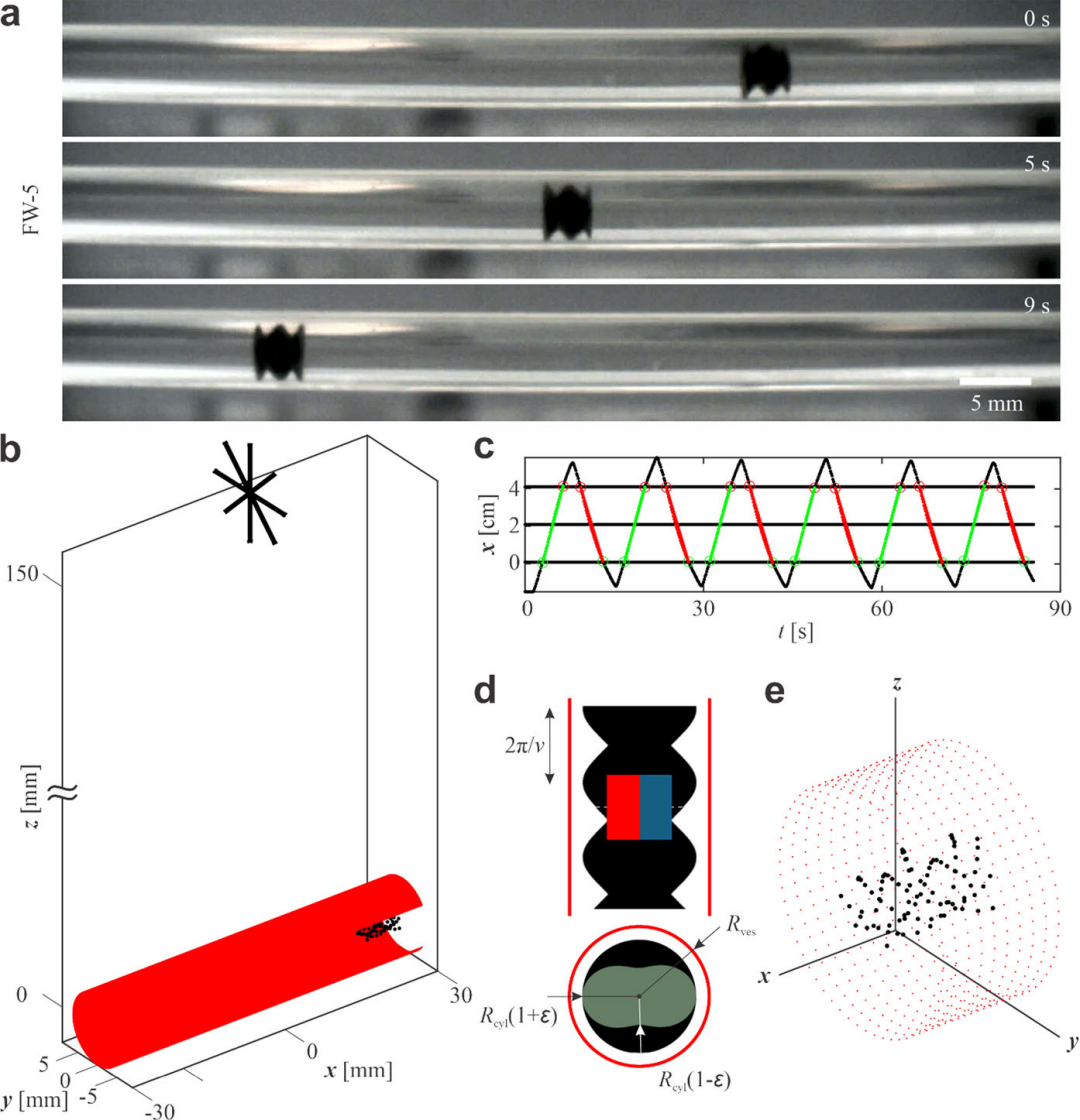
Phantom swimming experiments of untethered magnetic robots (UMRs) in water-filled horizontal vessels. **a** Side-view camera image showing the 1/2 in. horizontal vessel and the UMR FW-3 ($\nu =1.4$) swimming within it. **b** Schematic illustration of the experimental setup showing the magnetic moment of the rotating permanent magnet (RPM) (black arrows at $z=15$ cm), the horizontal soft-PVC vessel (red), and the UMR inside the vessel (black). The RPM generates a rotating magnetic field that actuates the UMR. **c** UMR trajectory over time, showing forward (green) and backward (red) swimming. Average position and boundary limits are indicated by thin horizontal lines. Propulsion speeds are calculated from the slope of the position-time plots. **d** Diagram showing the screw-shaped UMR (black) confined within a vessel of radius $R_{\textrm{ves}}$ (red lines), in both side and top views. Two embedded permanent magnets are aligned with their magnetic dipoles perpendicular to the UMR’s long axis. **e** Representation of the UMR and vessel wall using Stokeslet points. Black dots denote the UMR surface and red dots the vessel wall, enabling modeling of hydrodynamic interactions

In this study, we systematically investigate the swimming dynamics of screw-shaped UMRs with varying geometries in vessels of different diameters (Fig. 1). The primary objective is to identify UMR designs that maintain consistent and predictable propulsion across all tested confinements (Fig. 2). To this end, a broad selection of UMRs–differing in pitch, length, and other geometric parameters–is first tested using experiments conducted in artificial vessel models (phantoms). The most robust and consistent performers are subsequently validated *ex vivo* in sheep aorta and iliac artery models (Figs. 1c and d).

The remainder of the manuscript presents the phantom and *ex vivo* experimental setups, the design and fabrication methodology for UMRs, the theoretical modeling of propulsion speed, and a detailed discussion of the results. We conclude by summarizing the key findings and their implications for future biomedical applications involving controlled navigation of UMRs in confined environments.

## Materials and methods

We begin with a detailed overview of the design and fabrication processes for the UMRs. Two distinct groups of UMRs were developed and tested in vessels with varying diameters. We present the relevant design parameters for each group. This is followed by a description of the geometry, procedures and wireless magnetic manipulation setup used to evaluate the swimming performance of UMR in both phantom and *ex vivo* environments. We conclude the section by outlining the expected fluidic and structural effects that influence UMR locomotion under confinement.

### Design and fabrication of screw-shaped UMRs

**Table 1:** Parameters of the different untethered magnetic robots (UMRs) with fixed length (FL) used for swimming speed tests in artificial vessel models. Parameters are normalized wavenumber $\nu$, number of starts *N*, amplitude of starts $\epsilon$, total length $L_{\textrm{UMR}}$, minimum radius $R_{\textrm{min}}$, cylinder radius $R_{\textrm{cyl}}$, maximum radius $R_{\textrm{max}}$ and points per cross-section $N_{\textrm{c}}$

UMR FL-	1	2	3	4	5	6	7	8	9	10	11
$\nu$	0.25	0.5	1	1.25	1.4	1.55	1.8	2	2.33	3	3.5
*N*	2	2	2	2	2	2	2	2	2	2	2
$\epsilon$	0.33	0.33	0.33	0.33	0.33	0.33	0.33	0.33	0.33	0.33	0.33
$L_{\textrm{UMR}}$ [mm]	7.47	7.47	7.47	7.47	7.47	7.47	7.47	7.47	7.47	7.47	7.47
$R_{\textrm{max}}$ [mm]	2.08	2.08	2.08	2.08	2.08	2.08	2.08	2.08	2.08	2.08	2.08
$R_{\textrm{min}}$ [mm]	1.05	1.05	1.05	1.05	1.05	1.05	1.05	1.05	1.05	1.05	1.05
$N_{\textrm{c}}$	100	100	100	100	100	100	100	100	100	100	100

The rotating magnetic field $\textbf{B}(t)$ generated by the rotating permanent magnet (RPM) induces a torque $\varvec{\tau } = \textbf{m}_{\textrm{UMR}} \times \textbf{B}$ on the magnetic moment $\textbf{m}_{\textrm{UMR}}$ embedded within the UMR. When this magnetic torque exceeds the opposing resistive torques–arising from fluid–structure interactions and frictional contact with vessel walls–the UMR rotates about the same axis as the RPM. Consequently, the UMR’s geometry plays a critical role in determining its propulsion performance and swimming stability in response to the rotating magnetic field. Key influencing factors include fluid velocity, wall friction, vessel geometry, and internal flow resistance.

Each UMR is constructed as a screw-shaped rigid body with length $L_{\textrm{UMR}}$, maximum diameter $2R_{\textrm{max}}$, and a magnetic moment $\textbf{m}_{\textrm{UMR}}$ oriented perpendicular to its longitudinal axis (Fig. 2). The geometry is defined from a cross-sectional radius profile $\rho (\theta ) = R_{\textrm{cyl}}[1 + \epsilon \sin (N\theta )]$ that is rotated and translated along the *z*-axis to produce a helical shape with normalized pitch length $2\pi /\nu$. The complete surface of the UMR is thus described by:

1 $$\begin{aligned} \textbf{r}(\theta ,\zeta ) = \zeta \hat{\textbf{i}} + \rho (\theta )\left[ \cos (\nu ^{}\zeta + \theta )\hat{\textbf{j}} + \sin (\nu ^{}\zeta + \theta )\hat{\textbf{k}}\right] , \end{aligned}$$

where $\theta \in [0, 2\pi )$ and $\zeta \in [0, L_{\textrm{UMR}}]$ are helical coordinates. The modulated radius $\rho (\theta )$ describes the cross-sectional perturbation, with amplitude $\epsilon$ and number of starts *N*. All UMRs were fabricated with a cylinder radius $R_{\textrm{cyl}} = 1.56$ mm, amplitude $\epsilon = 0.33$, and $N = 2$ starts. The resulting radius ranges from $R_{\textrm{min}} = R_{\textrm{cyl}}(1 - \epsilon )$ to $R_{\textrm{max}} = R_{\textrm{cyl}}(1 + \epsilon )$. The wavenumber $\nu ^{} = 2\pi /\lambda$ relates to wavelength $\lambda$, and the normalized wavenumber is $\nu = \nu ^{}R_{\textrm{cyl}}$. The number of helical turns is defined as $N_{\textrm{t}} = L_{\textrm{UMR}}/\lambda = \nu L_{\textrm{UMR}}/(2\pi R_{\textrm{cyl}})$. The degree of confinement is characterized by the ratio $L = R_{\textrm{ves}}/R_{\textrm{cyl}}$.

Two UMR groups were tested: the FL group, where all robots share the same length $L_{\textrm{UMR}}$ but differ in $\nu$ (ranging from 0.25 to 3.5), and the FW group, where each design has a single helical turn ($N_{\textrm{t}} = 1$) and the pitch length varies inversely with $\nu$ (Tables 1 and 2, Fig. 1a). In the FW group, UMR length decreases with increasing $\nu$. The design dimensions like UMR diameter and length were based on anatomical constraints like aorta and iliac artery diameter. Previous studies use similar dimensions for UMR [14]. The lower limit is $\nu$ = 0.25 for FL-group, because then the shape is only a part of helical shape and actuation becomes unstable. The upper limit of normalized wavenumber $\nu$ at 3.5 is given by fabrication limits due to too shallow angles when 3D printing. For the fixed wave group, $\nu$ could not be reduced further because then the UMR becomes too long.

UMR geometries were generated in MATLAB (MathWorks, Natick, USA) and exported as STL files. For magnet integration, the STL files were modified in SolidWorks (Dassault Systèmes) to include internal cut-outs for housing permanent magnets. The UMR design consists of two mirrored halves that are joined together to enclose the magnets. The UMR diameter was chosen to ensure close-fitting operation within 3/16-inch diameter vessels, while the length was selected to maintain maneuverability and match dimensions used in prior studies [38, 39].

Fabrication of UMRs was performed using Masked Stereolithography Apparatus with a Phrozen Sonic Mini 4K printer and Phrozen Aqua-Gray 4K resin. A layer height of 50 $\upmu$m was used. Printed parts were cleaned for 7 min in an ultrasonic bath filled with isopropyl alcohol (IPA), followed by 12 min of post-curing in an Elegoo Mercury Plus curing station. Final UMRs had lengths ranging from 4 to 10 mm and diameters of approximately 4.16 mm. Each UMR contained two embedded cubic permanent magnets (1 mm per side) made of NdBFe Grade-N45 (S-01-01-N, Supermagnete, Gottmadingen, Germany) with a magnetic moment of $8.4 \times 10^{-4}$ A$\cdot$m$^{2}$.

**Table 2:** Parameters of the UMR group with fixed wave (FW, $N_t = 1$) used for swimming speed tests in artificial vessel models. The parameters are normalized wavenumber $\nu$, number of starts *N*, amplitude of starts $\epsilon$, number of turns $N_{\textrm{t}}$, total length $L_{\textrm{UMR}}$, minimum radius $R_{\textrm{min}}$, cylinder radius $R_{\textrm{cyl}}$, maximum radius $R_{\textrm{max}}$ and Points per cross-section $N_{\textrm{c}}$

UMR FW-	1	2	3	4	5	**6 **
$\nu$	1	1.25	1.4	1.55	2	2.33
*N*	2	2	2	2	2	2
$\epsilon$	0.33	0.33	0.33	0.33	0.33	0.33
$N_{\textrm{t}}$	1	1	1	1	1	1
$L_{\textrm{UMR}}$ [mm]	9.73	7.77	6.88	6.29	4.82	4.13
$R_{\textrm{min}}$ [mm]	1.05	1.05	1.05	1.05	1.05	1.05
$R_{\textrm{cyl}}$ [mm]	1.56	1.56	1.56	1.56	1.56	1.56
$R_{\textrm{max}}$ [mm]	2.08	2.08	2.08	2.08	2.08	2.08
$N_{\textrm{c}}$	100	100	100	100	100	100

### Phantom experimental setup for UMR swimming

The experimental setup consisted of a water-filled vessel, horizontally clamped to a table, with a camera mounted on the same table to track the position of the UMR (Fig. 2). An RPM was placed was placed 15 cm above the center of the vessel to generate a rotating magnetic field $\textbf{B}(t)$ (schematically illustrated in Fig. 2a). The RPM position is stationary at constant x-position during the experiment. The 15 cm separation distance between the RPM and the vessel was chosen to ensure a nearly uniform, gradient-free magnetic field at the location of the UMR. This setup enables us to isolate and study torque-driven locomotion without significant influence from pulling magnetic forces. Additionally, this distance reflects clinically relevant conditions, where external magnetic sources would be positioned outside the patient’s body at similar separations from target vessels. The 15 cm distance represents a practical trade-off that ensures a sufficiently uniform magnetic field for torque-driven locomotion while reflecting realistic anatomical constraints in potential clinical scenarios. The RPM was constructed from NdBFe Grade-N45 material in a cylindrical shape (35 mm diameter, 20 mm height) and had a magnetic moment of 18.89 A$\cdot$m$^{2}$. At the specified height of 15 cm, the resulting magnetic flux density was measured to be 1.2 mT which provides sufficient torque for propulsion.

The rotational motion of the RPM was driven by a Maxon 18 V brushless DC motor, while its orientation was precisely controlled using a KUKA 6-degree-of-freedom robotic manipulator (KUKA KR-10 1100-2, KUKA, Augsburg, Germany). The axis of RPM rotation was aligned parallel to the longitudinal axis of the vessel. The RPM was rotated at a constant frequency of 10 Hz. The 10 Hz actuation frequency was selected to ensure that all UMR designs operate below their step-out frequency, enabling consistent propulsion without magnetic desynchronization. The step-out frequency for each design was characterized by measuring the average swimming speed over a range of actuation frequencies. This approach guarantees that performance comparisons are made within the synchronized propulsion regime across all tested UMRs. The selected values of 15 cm distance, 1.2 mT magnetic flux density and 10 Hz actuation frequency were informed by prior experimental characterization and design constraints. As detailed in our previous work [40], the chosen frequency ensures that all tested UMR designs operate below their step-out thresholds, which were experimentally determined by measuring swimming speeds over a range of actuation frequencies. Step-out frequency is dependent on UMR length, decreases for longer UMRs. For 9 mm long UMR, a step-out frequency of 27Hz was found. Below step-out frequency, speed and frequency are linearly related as found previously by simulation and experiments. Therefore, frequency should have no effect on optimal design selection and was fixed to 10 Hz. Prior to swimming trials, the UMR was carefully placed inside the vessel without air bubbles and guided to the starting position using a secondary permanent magnet.

During operation, the UMRs swam below the RPM within a vertical range of $z = -2.5$ to $z = +2.5$ cm relative to the RPM’s center (Fig. 2b). Each time the UMR passed a predefined boundary, the RPM’s rotational direction was reversed, causing the UMR to swim in the opposite direction. Each UMR from Tables 1 and 2 performed at least four round-trip swims between boundaries (Fig. 2c). The robots tested belonged to two design groups: fixed-length (FL) and fixed-wave (FW). The FL group was designed to isolate the effect of helical pitch on swimming performance while keeping the overall body length constant–allowing analysis of how changes in wavenumber affect propulsion in vessels of varying diameters. Conversely, the FW group was constructed to maintain a single helical turn across all designs, enabling the study of how total robot length influences swimming dynamics when the pitch length is varied. This dual-group approach provides a comprehensive understanding of how different geometric parameters independently affect propulsion efficiency and stability under confinement.

To simulate physiological variation in vessel diameter, four phthalate-free soft-PVC vessel models with inner diameters of 3/16 in., 1/4 in., 3/8 in., and 1/2 in. were used. This range of vessel diameters were based on anatomical constraints like aorta and iliac artery diameter. The camera captured video at 30 frames/s with a resolution of 1280$\times$1024 pixels. These recordings were analyzed using Open Source Physics tracker software to extract the frame-by-frame positions of the UMRs. Data were further processed and visualized using MATLAB. The UMR designs exhibiting the most consistent swimming performance across vessel sizes were selected for subsequent *ex vivo* evaluation.

### *Ex Vivo* trials in sheep aorta and iliac arteries

For the *ex vivo* experiments, UMRs were coated using LipoCoat 4AC coating technology via manual dip-coating for 10 s, followed by overnight drying under ambient conditions in the dark. This lipid-based coating plays a crucial role in enhancing hemocompatibility by minimizing interactions between the robot surface and blood components. As demonstrated in prior studies [40], LipoCoat 4AC significantly reduces fibrinogen adsorption by $95\%$ and bacterial attachment by over $99\%$ compared to uncoated materials. Moreover, it delays fibrin formation by more than six minutes and prevents complement activation and platelet aggregation, thereby lowering the risk of clotting, inflammation, and infection. These properties are critical for ensuring the safe deployment of UMRs in blood-contacting environments and represent an important step toward their clinical translation. Biological samples–specifically the aorta and iliac arteries–were harvested from a euthanized sheep (Fig. 1a). Euthanasia was performed using 15 mL of Euthasol (AST Pharma, Oudewater, the Netherlands) mixed with 5 mL of Heparin (5000IU/mL, Leo Pharma, Ballerup, Denmark). A total of 800 mL of the sheep’s blood was collected and used for perfusion.

During the experiments, 500 mL of collected blood was circulated through the harvested arteries at a minimal flow rate to prevent vessel collapse while maintaining physiological conditions. The flow direction is from aorta to iliac artery. To enable real-time visualization of the vascular anatomy and UMR motion, 30 mL of Iomeron (300 mg/mL iodine-based contrast agent) was added to the blood reservoir to enhance vessel radiopacity during fluoroscopy. Wireless magnetic actuation of the UMRs was carried out using the same RPM setup described in the phantom experiments. All procedures were conducted under a clinical-grade C-Arm fluoroscopy system (Siemens Healthineers Artis Pheno, Erlangen, Germany), which provided continuous X-ray imaging at 5 frames per second. The fluoroscopy system was operated using pulsed mode with a tube voltage of 70–80 kVp and a dose-per-frame of approximately 0.2–0.3 mGy, resulting in a total estimated dose of 30–45 mGy per 3-minute acquisition, depending on duration and imaging settings. This radiation exposure is well within acceptable limits for preclinical studies and allows high-contrast tracking of the embedded permanent magnets in the UMR.

To facilitate spatial analysis, selected frames from fluoroscopic recordings were exported and processed using clinical DICOM-compatible imaging software. These images were aligned and stitched to visualize the complete vascular geometry, including anatomical bifurcations such as the LCIA, RCIA, and MSA. This composite imaging approach enabled accurate assessment of UMR position and motion trajectories within the vascular phantom, supporting both qualitative and quantitative evaluation of navigation performance.

The RPM was positioned directly above the midpoint of the aorta, with an actuation frequency of 10 Hz and a vertical separation of 15 cm between the RPM and arterial tissue. UMR motion was recorded at 5 frames per second using fluoroscopy imaging (Fig. 1b). The two most consistent swimmers identified during testing in artificial vessel models—FL-9 and FW-5 from the fixed-length and fixed-wave groups, respectively—were selected for evaluation in the ex vivo model.

### Theoretical estimation of UMR translational velocity using the stokeslet method

The translational velocity $v_{\textrm{Stokeslet}}$ of UMRs inside cylindrical vessels of radius $R_{\textrm{ves}}$ is estimated by applying the method of regularized Stokeslets [41]. Stokeslet points are distributed along the surface of the screw-shaped UMR, as defined by Equation (1), and along the inner surface of the vessel (Fig. 2e). Each Stokeslet point on the UMR surface is assigned a velocity given by $\textbf{v}(\theta , \zeta ) = \omega \hat{\textbf{z}} \times \textbf{r} + v_{\textrm{Stokeslet}}\hat{\textbf{z}}$, where the first term represents rotational motion due to angular velocity $\omega$ and the second term corresponds to translational propulsion. In contrast, Stokeslet points on the vessel wall are held stationary, simulating a no-slip boundary condition.

The forces acting on all Stokeslet points are computed, and the net hydrodynamic force acting on the UMR is determined. The translational velocity $v_{\textrm{Stokeslet}}$ is then iteratively adjusted until this net force reaches zero, ensuring steady-state propulsion. This simulation is performed across a range of normalized wavenumbers $\nu$ and confinement ratios $1/L = R_{\textrm{cyl}}/R_{\textrm{ves}}$. For the current study, UMRs were modeled with a perturbation amplitude $\epsilon = 0.33$ and normalized wavenumber values spanning $\nu = R_{\textrm{cyl}}\nu ^* = 1$ to 2.33. The confinement ratio $R_{\textrm{cyl}}/R_{\textrm{ves}}$ ranged from 0.12 (small vessels) to 0.33 (large vessels), as shown in Fig. 4.

Since the theoretical model does not account for surface friction, wall contact, or off-center swimming effects, its predictions ($v_{\textrm{Stokeslet}}$) are compared to experimentally measured average velocities ($v_{\textrm{avg}}$). Off-center swimming is particularly difficult to model analytically because it introduces asymmetrical flow fields, variable hydrodynamic drag, and intermittent wall interactions that are sensitive to minor variations in robot orientation and vessel geometry. These effects result in non-linear and spatially varying forces that are not captured by idealized models assuming axisymmetric, centered locomotion. To bridge this discrepancy, a correction model is introduced:

2 $$\begin{aligned} v_{\textrm{fit}} = p_0 v_{\textrm{Stokeslet}} - p_{\textrm{ves}} R_{\textrm{ves}} - p_{\textrm{turn}} N_{\textrm{t}} - p_{\textrm{L}} / L_{\textrm{UMR}}, \end{aligned}$$

where $p_0$ is a scaling parameter, $p_{\textrm{ves}}$ accounts for vessel diameter effects, $p_{\textrm{turn}}$ adjusts for the number of helical turns $N_{\textrm{t}}$, and $p_{\textrm{L}}$ reflects the influence of UMR length. The best-fit parameters are determined by minimizing the error function $\chi = \sqrt{\langle (v_{\textrm{fit}} - v_{\textrm{avg}})^2 \rangle }$, thereby aligning the theoretical predictions with experimental observations.

## Results

**Fig. 3:**
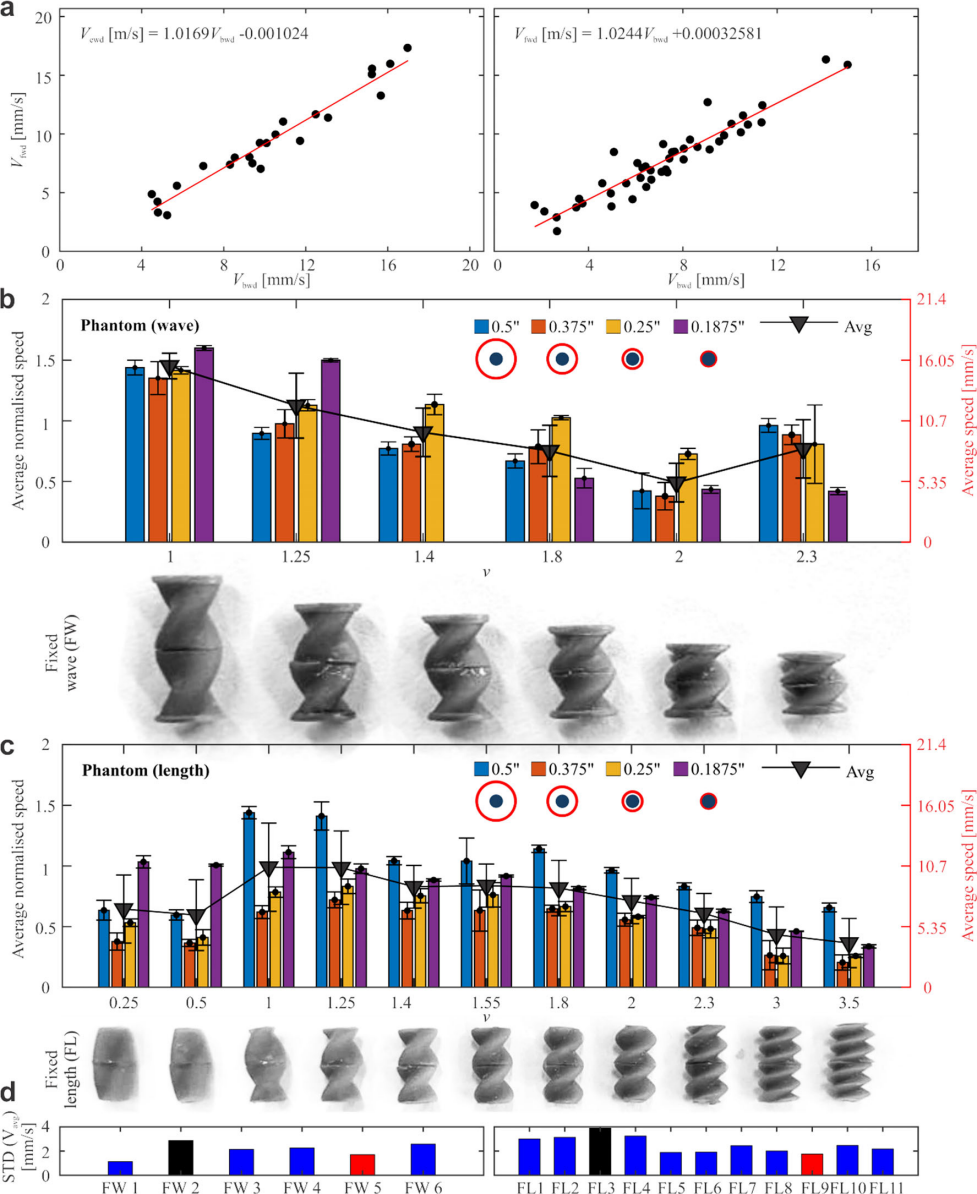
Selection of the most and least consistent swimmers in groups FW and FL. **a** Forward and backward swimming speeds are linearly related with slope $\approx 1$ and near-zero intercepts for both groups FW (left) and FL (right). **b**–**c** Average normalized speeds across different vessel diameters during phantom experiments for FW (**b**) and FL (**c**) designs. **d** Standard deviation of speed for FW (left) and FL (right). FW-5 and FL-9 (red) are the most consistent swimmers, showing low speed and variability. **(Movies S1, S3, S5, S7, S9, S11, S13, S15)** for different vessel size. FW-2 and FL-3 (black) are the least consistent, with high speed and variability. **(Movies S2, S4, S6, S8, S10, S12, S14, S16)** for different vessel size

### Swimming Performance of UMRs in Artificial Vessel Models

The forward and backward swimming velocities of UMRs from the FL and FW groups were determined across various vessel diameters by tracking the UMR position over time, as illustrated in movies **S1-S16** and Fig. 2c. Forward and backward velocities showed a strong linear correlation, as depicted in Fig. 3a. The rotating magnetic field by the RPM initiates a torque on the UMR which together with the helical shape leads to similar forward propulsion for both rotational directions of the RPM and UMR. There are other forces which could have effects on the propulsion. The magnet in the UMR also leads to gradient forces from the gradient of the RPM. These forces were not considered because the magnetic field gradient is small at height 15 cm and horizontal distance $\pm 2.5$ cm from the RPM and on time average they are zero. Furthermore, they act on all UMRs equally. Also, UMRs didn’t show any movement for stationary RPM, so gradient forces are too weak to initiate UMR motion. Gravity had an effect on UMR motion since the UMRs are mainly at the lower vessel surface. This could be improved in the future by density matching of UMR more closely to water or blood, respectively. Contact with vessel surface could lead to adhesion forces. These forces could be minimized by non-adhesive UMR coating.

Experimental observations revealed that UMRs achieved their highest swimming speeds in both large and small vessels when the normalized wavenumber $\nu$ was approximately unity. In the FW group, average swimming speed decreased with decreasing UMR length or increasing $\nu$ (Fig. 3b). For the FL group, speed was relatively uniform across designs in intermediate vessel diameters, whereas this trend was not evident for the FW group (Fig. 3c).

Among the FW designs, FW-5 exhibited the lowest average speed along with one of the lowest standard deviations, and was thus identified as the most stable swimmer (Fig. 3d). Conversely, FW-2 demonstrated one of the highest average speeds but also the highest standard deviation, marking it as the most unstable swimmer in the FW group.

For the FL group, the highest swimming speeds were observed in the smallest and largest vessels, while intermediate vessel diameters yielded lower speeds. The average speed across all vessel sizes peaked around $\nu = 1$. FL-9 demonstrated the lowest average speed and smallest standard deviation, indicating highly stable motion. In contrast, FL-3 showed the highest average speed and largest standard deviation, reflecting a more unstable swimming behavior.

**Fig. 4:**
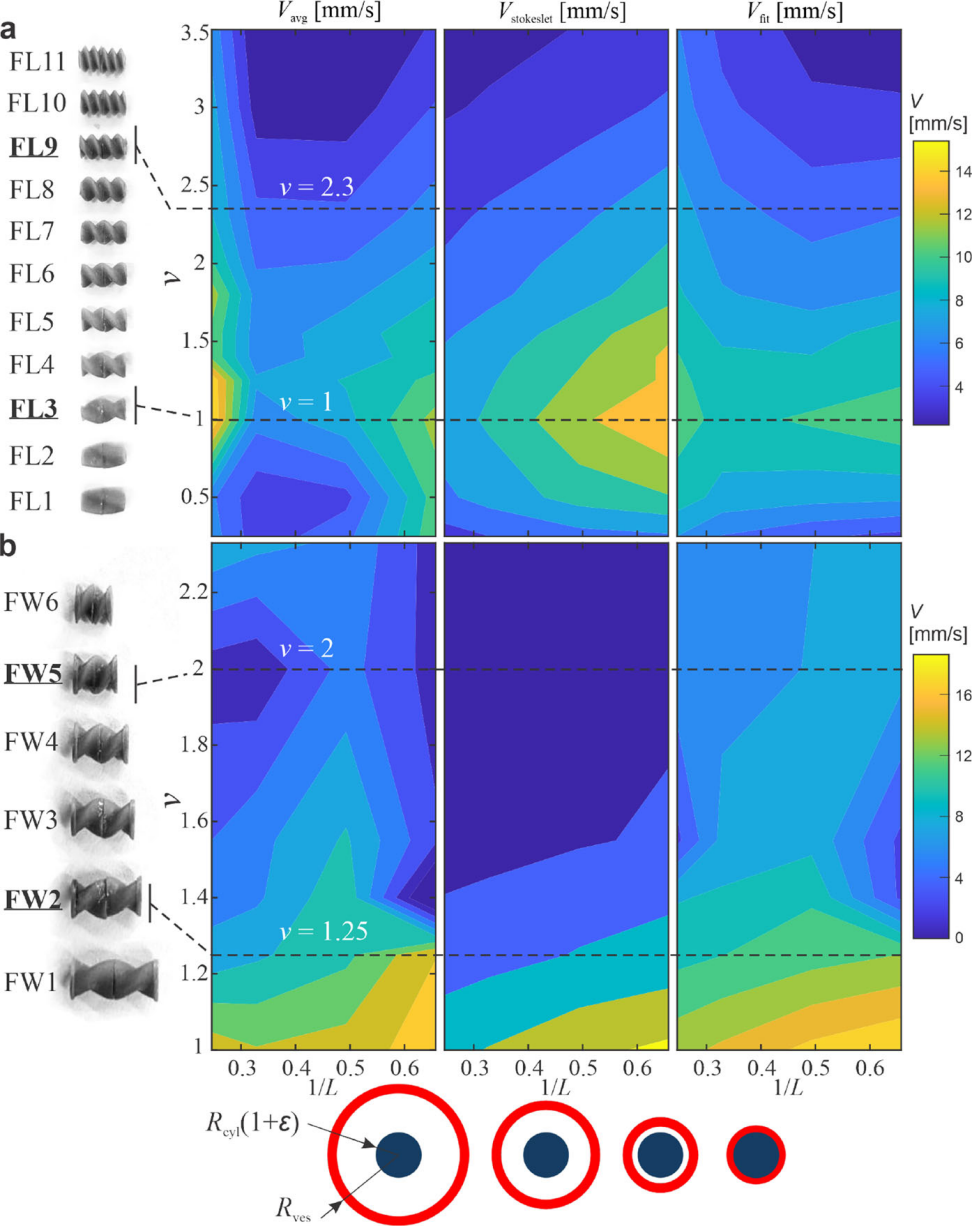
Surface plots of velocity of UMRs showing speed of design versus vessel size. UMR design is represented by normalized wavenumber $\nu$. Vessel size is represented by ratio of UMR radius and vessel radius $1/L=R_{\textrm{cyl}}/R_{\textrm{ves}}$. Results of UMRs with a) fixed length (FL) and b) fixed wave (FW) are shown for phantom experiments (left), simulations using Stokeslet model (middle) and Stokeslet model with friction due to wavenumber and vessel walls. Most robust swimmers are FL-9 and FW-5 and swimmers with variable speed are FL-3 and FW-2

Experimentally measured swimming speeds are presented as contour plots in Fig. 4. The highest velocities were observed at a normalized wavenumber of $\nu \approx 1$. Within the FL group, speeds were greater in the smallest and largest vessel diameters, while intermediate vessels yielded lower propulsion speeds. In contrast, FW group robots exhibited increasing speed in tighter vessels, again peaking around $\nu \approx 1$.

For comparison, simulation results based on the Stokeslet method are shown alongside the experimental data. In the model, swimming speed increases as vessel diameter decreases and as $\nu$ approaches unity (middle in Figs. 4a and b). The averaged experimental and simulated velocities are visualized as surface plots. Discrepancies between measured and modeled speeds can be attributed to several factors, including wall interactions, off-center swimming due to gravity, and minor misalignments in the rotational axis.

Interestingly, for the FL group, the highest experimental speeds occurred in the largest vessels, suggesting that wall contact may hinder propulsion in smaller vessels–an effect not captured by the idealized model. For the FW group, swimming speed generally increased with longer pitch lengths (lower $\nu$) and narrower vessels.

These trends reveal that UMRs with different pitches respond distinctly to varying vessel sizes. High-pitch UMRs (low $\nu$) perform optimally in narrow vessels. Intermediate-pitch robots can achieve high speeds but exhibit more variability across vessel sizes. Low-pitch UMRs swim slowly but demonstrate consistent behavior regardless of the confinement, indicating reduced sensitivity to environmental variation.

**Table 3:** Results of fitting experimentally measured UMR speeds by predicted speeds using Stokeslets method and further taken into account influences by total length $L_{\textrm{UMR}}$ of UMR, normalized wavenumber $\nu$ of UMR and vessel radius $R_{\textrm{ves}}$. The fitting eq. is $v_{\textrm{fit}}=p_0 v_{\textrm{Stokeslet}} - p_{\textrm{ves}} R_{\textrm{ves}}- p_{\textrm{turn}} N_{\textrm{t}} - p_{\textrm{L}}/L_{\textrm{UMR}}$. The error function is $\chi =\sqrt{<(v_{\textrm{fit}}-v_{\textrm{avg}})^2>}$

Parameters	$p_0$	$p_{\textrm{ves}}$ [1/s]	$p_{\textrm{turn}}$ [$\upmu$m/s]	$p_{\textrm{L}}$ [mm$^2$/s]	$\chi$ [mm/s]
group FL (no fit)	1	0	0	0	3.3
group FL (best fit)	0.81	-1.2855	$-213$	37	2.2
group FW (no fit)	1	0	0	0	6.0
group FW (best fit)	1.55	0.0021	$-4076$	5.5	2.1

The error between the measured swimming speed and the speed predicted by the Stokeslet method is substantial, averaging 3.3 mm/s for the FL group and 6 mm/s for the FW group (Table 3, Fig. 4). When additional correction terms are applied as described in Eq. 2–accounting for vessel size, number of helical turns, and UMR length–the average error decreases to 2.2 mm/s for the FL group and 2.1 µm/s for the FW group.

The scaling factor $p_0$ varies between being less than and greater than one, indicating that the Stokeslet model alternately overestimates or underestimates the experimental speeds. The vessel size correction term has a stronger effect for the FL group, with its negative sign suggesting that swimming speed increases with vessel diameter more than predicted by the theoretical model. The number of helical turns has a more pronounced impact in the FW group, and the negative sign of $p_{\textrm{turn}}$ implies that the experimental speed increases with turn count beyond what the Stokeslet model anticipates. UMR length shows a greater influence in the FL group; as length increases, smaller corrections are required to match experimental speed. For shorter UMRs, the Stokeslet method tends to overpredict the swimming speed.

The corrected velocity $v_{\textrm{fit}}$ is visualized as a contour plot in Fig. 4, showing improved agreement with the experimentally measured average velocity $v_{\textrm{avg}}$ compared to the original Stokeslet prediction $v_{\textrm{Stokeslet}}$.

The discrepancies between the theoretical predictions based on the Stokeslet method and the experimental results, particularly in the FW group, highlight important limitations in the predictive capability of the idealized model. The discrepancies observed–especially in the FW group–are primarily due to the ideal assumptions embedded in the Stokeslet formulation, such as neglecting off-center trajectories, wall interactions, and adhesion effects. These limitations become more pronounced in the FW group, where the shorter UMR designs exhibit greater instability and susceptibility to local hydrodynamic perturbations, leading to increased tumbling and lateral drift. Our findings clearly show that velocity deviations are strongly affected by these nonlinear phenomena that are not captured in a linear superposition of Stokeslets. To address these limitations, we introduced a linear correction model (eq. 2) incorporating four parameters: vessel diameter, number of helical turns, UMR length, and a scaling factor. This hybrid model reduced the average prediction error from 3.3 mm/s to 2.2 mm/s in the FL group and from 6.0 mm/s to 2.1 mm/s in the FW group, significantly improving agreement with experimental data while retaining a tractable analytical form. Although simplified, this modeling approach retains conceptual and comparative value by revealing key parametric dependencies (e.g., the role of pitch, turns, and length) and enabling efficient screening of design trends over a broad space. It thus serves as a useful tool for initial robot design selection, even if precise quantitative prediction is limited. More advanced computational methods, such as full computational fluid dynamics (CFD), could offer better accuracy in modeling confined swimming with strong wall effects and off-axis trajectories. However, these methods come with substantial trade-offs. First, CFD simulations of torque-driven helical propulsion in long and narrow cylindrical domains are computationally expensive and often limited to steady-state or idealized scenarios with fixed boundary conditions. Secondly, as shown in our experimental work, the UMRs frequently exhibit nonlinear behaviors–such as intermittent contact with the wall, local adhesion, off-center wobbling, and tumbling–which are difficult to model deterministically, particularly when working with clinically sized geometries, real vessel compliance, and flow disturbances. These effects introduce statistical variability that a deterministic CFD model may not capture effectively. Additionally, the robot itself perturbs the local flow profile significantly due to its scale relative to the vessel, violating assumptions of many boundary-layer or creeping-flow approximations. In this context, we prioritized a framework that allows for rapid prototyping and qualitative insight across a design spectrum, supported by empirical correction terms. Our approach complements high-fidelity modeling rather than competing with it, and may serve as a foundation for future efforts incorporating hybrid analytical-numerical models or machine learning–assisted surrogate models trained on CFD outputs.

With the data gathered, we can determine the most and least consistent all-round swimmer inside arbitrary confinement to test their swimming in aorta and iliac artery. As shown in Fig. 3, except for very low values of $\nu$ there is very little correlation between swimming speed and the size of confinement. In Fig. 3 can be seen that in all measurements a UMR of $\nu \approx$1 swims faster than other UMR designs. So based on that we can conclude the best performing UMR in terms of speed is the $\nu \approx$ 1 UMR. On the other hand speed variation and standard deviation decreases as $\nu$ increases and therefore indicating higher stability / motion robustness. The most robust swimmers were deemed to be the $\nu =2.33$ for FL (FL-9) and $\nu =2$ for FW group (FW-5), respectively. The irregular swimmers were $\nu =1$ for FL (FL-3) and $\nu =1.25$ for FW (FW-2), respectively.

### Swimming in *ex vivo* confined spaces by steering from the Aorta into the Iliac Artery

**Fig. 5:**
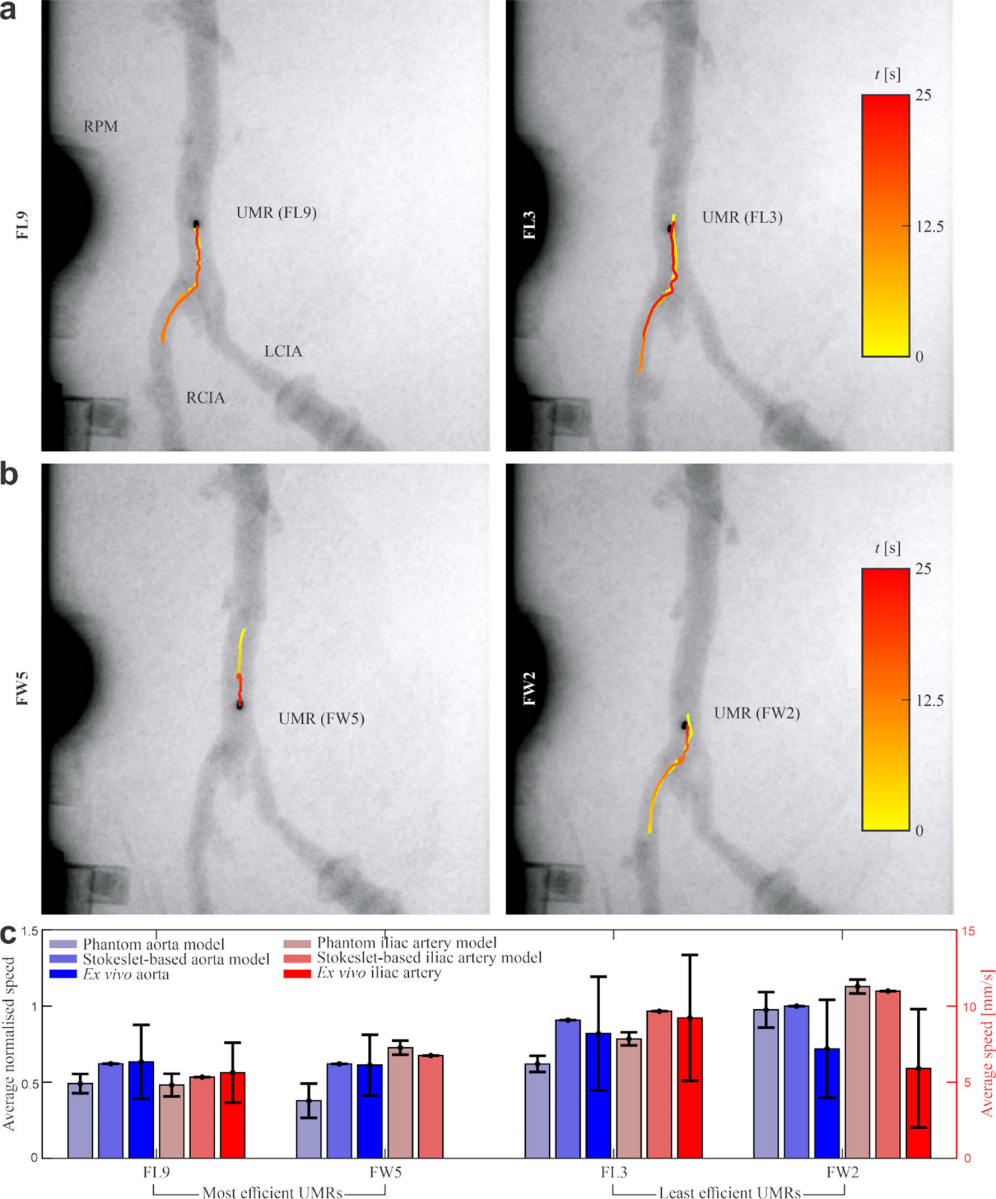
The sheep aorta bifurcates into the left and right iliac arteries. The cubic permanent magnet inside the UMR (1 mm side) appears as a dark spot in the fluoroscopy image, indicating its position. Starting in the aorta at $t=0$ s, the UMR moves through the bifurcation into the left iliac artery, then returns to the aorta–demonstrating controlled, repeatable navigation for UMRs a) FL-9 **(Movie S17)** and FL-3 **(Movie S18)** from fixed length group FL and b) FW-5 **(Movie S19)** and FW-2 **(Movie S20)** from fixed wave group FW. c) UMR speeds were tracked upstream and downstream in both the aorta and iliac artery. FL-9 and FW-5 showed higher and more variable speeds in anatomical vessels compared to straight channels. FL-3 displayed comparable speeds across all regions. FW-2 showed reduced speed in the aorta and no motion in the iliac artery

The main goal of the *ex vivo* experiments was to validate the phantom experiment findings in an ex vivo environment, to see if the found relation holds. For FW- and FL-groups, the most consistent and least consistent swimming UMR-designs were selected for further investigations of swimming in the ex vivo set-up consisting of vessels aorta and iliac artery (Figs. 5a and b and supplementary movies **S17-S20**). Actuation begins in the aorta and the UMR moves downstream under influence of flow and induced trust by external magnetic field rotation. At the bifurcation point it enters the left iliac artery and moves some distance. After this the UMR returns to the bifurcation point against the stream and continues to move the aorta upstream. We averaged the velocity for upstream and downstream motion to cancel out the effect of low flowrate since flowrate was not the point of our research. The UMR moved in both directions (forward and backward) both in large vessel aorta and in small vessel iliac artery. Due to experimental time constraints in clinical setting, the maneuvering was only conducted in one iliac artery as representation of small vessels. In future, maneuvering in both iliac arteries should be demonstrated.

The results of the *ex vivo* trial are presented in Fig.  5c. The highest speeds $8.8-9.9$ mm/s were found for FL-3. UMR FW-2 has intemediate speeds $6.3-7.7$ mm/s. The lowest range was found for FL-9, with a speed range $6.0-6.8$ mm/s. The FW-5 design moved $4-7$ mm/s in aorta, but did not swim in the iliac artery because it kept getting stuck. This is most likely due to the ratio $R_{\textrm{cyl}}/L_{\textrm{UMR}}\approx 1$ causing it to more easily tumble instead of keeping parallel with the walls of the blood vessel. The standard deviation of FL-9 is 38.15$\%$ and 34.98$\%$ for aorta and iliac artery respectively. The rest of the standard deviations are: 44.75$\%$ and 65.76$\%$ for FW-2, 45.63$\%$ and 44.78$\%$ for FL-3, and 32.61$\%$ for FW-5. Between FL-3 and FW-5 the standard deviation decreased 46.61$\%$. Comparison between experiments conducted in artificial vessel models and those performed in ex vivo tissue is possible, although the former were conducted in water, whereas the latter used blood Usually, the effect of Non-Newtonian fluid instead of Newtonian fluid on speed is not very large and it is the same factor for all UMRs $v/v_N=(1+\beta _S De^2)/(1+De^2)$, where *v* and $v_N$ are speed in non-Newtonian liquid and speed in Newtonian liquid, respectively, $b_S$ and Deborah number *De* are non-Newtonian fluid properties (see Lauga 2007 [42] and Li 2015 [43] ). It is expected that UMR shape and vessel diameter have larger effects. Comparing the swimming speed between aorta and iliac artery: FL-9 decreased by 11.30$\%$, FW-2 by 17.87$\%$, and FL-3 by $-$12.56$\%$. In all cases, standard deviation increases in the ex vivo experiments compared to the artificial vessel model experiments by 280is probably due to the more irregular aorta size compared to the straight vessels. Speed increases by $29\%$, $117\%$, $16\%$ and $-37\%$ for FL-9, FL-3, FW-2 and FW-5, respectively. Furthermore, ex vivo results in the iliac artery are compared to results obtained using the 1/4” artificial vessel model. Standard deviation increases by $164\%$, $795\%$ and $808\%$ for FL-9, FL-3 and FW-2, respectively. Speed increases by $17\%$, $27\%$ and $-25\%$ for FL-9, FL-3 and FW-2, respectively. Results of Stokeslet model are within the range of *ex vivo* measurements except for FW-2 iliac artery. The most consistent swimmer in aorta and iliac artery is FL-9 with medium speed in both arteries and lowest standard deviation. Both most consistent swimmers FL-9 and FW-5 have smaller velocities and smaller standard deviations than the less consistent swimmers. This confirms the observations from phantom model measurements.

Robustness was primarily defined based on low standard deviation in swimming speed across vessels of varying diameters, as this reflects minimal sensitivity to environmental changes–important for simplifying control strategies and ensuring safer actuation. FL-9 and FW-5 were selected accordingly. However, this criterion does not fully capture directional stability or susceptibility to stalling/tumbling, which became apparent in the more complex *ex vivo* environment. Future definitions of robustness should incorporate these dynamic stability factors for more comprehensive evaluation.

The swimming failure of FW-5 in the *ex vivo* iliac artery cannot be attributed solely to vessel geometry. FW-5, being the shortest design tested, is more susceptible to imperfections in the vascular wall–such as side branches or surface roughness–which can induce tipping or stalling, as seen in the videos. These interactions are particularly critical in complex anatomical environments and are not captured by idealized models. While blood perfusion, coating, and magnetic alignment were consistent across tests, the observed failure highlights the importance of considering surface irregularities and local anatomical features when evaluating UMR robustness.

## Conclusions

This study investigated the swimming performance of UMRs in vessels of varying diameters, followed by validation in an *ex vivo* model using harvested aorta and iliac arteries. While theoretical models predict enhanced swimming speed in narrower vessels due to confinement effects, experimental results showed that higher speeds often occurred in wider vessels. This discrepancy is attributed to wall interactions not captured in the model, which assumes ideal, centered swimming. In practice, UMRs often swim asymmetrically, leading to increased friction and hydrodynamic drag in narrower vessels. Prior experimental efforts have provided valuable insights that informed the overall design of this study. Our experiments are different in the actuation by a clinical more practical single rotating permanent magnet instead of Helmholtz setup for magnetic field generation, screw shape instead of helical filament, shape with incorporation of 2 magnets in the UMR for rotation stabilisation instead of a single magnet at the head, test of UMRs in *ex vivo* setups with blood as surrounding medium instead of water. This made it necessary to repeat experimental observation of swimming speed for different UMRs in narrow and wide vessels. Ideal Stokeslet method was extended by linear corrections to account for asymmetrical swimming, friction with vessel walls and tumbling.

UMRs in the FW group demonstrated that longer designs exhibited higher speed in smaller vessels, benefitting from enhanced guidance. As the UMRs shortened (i.e., as the normalized wavenumber $\nu$ increased), speed was larger in smaller vessels, with optimal swimming observed at medium diameters for $\nu$ values between 1.4 and 2. The shortest FW designs achieved their highest speeds in the widest vessels. Lowest speed and low variation of speed range was found for FW-5 with $\nu$ value of 2. In the FL group, swimming speed typically increased with $\nu$ up to a peak (typically between $\nu = 1$ and 1.8), then declined, showing that the relationship between geometry and propulsion is non-monotonic. Largest speeds are found for widest and narrowest vessel, whereas intermediate vessels had lower speeds. Lowest speed and low variation of speed range was found for FL-9 with $\nu$ value of 2.3.

The normalized wavenumber had a clear influence on both swimming speed and stability. Maximum propulsion was achieved at $\nu \approx 1$, while higher $\nu$ values contributed to greater rotational stability.

Despite these insights, a direct correlation between swimming speeds in the artificial vessel model and the ex vivo setup was not consistently observed. While stable swimmers such as FL-9 and FW-5 exhibited lower average speed and standard deviation as consistent behavior across both environments, unstable swimmers like FL-3 and FW-2 did not. Stable designs were sometimes unable to navigate confined vessels, as seen with FW-5 in the iliac artery (narrowest vessel). These findings emphasize the importance of UMR geometry–particularly normalized wavenumber and body length–in optimizing propulsion efficiency, swimming stability, and adaptability to anatomical variability. Future designs should leverage this understanding to improve reliability in clinical environments.

## Supplementary information

The following movies of UMRs are provided as supplementary information:

- **S1**: UMR FL-9 swimming inside a 0.5-inch diameter tube at frequency of 10 Hz.
- **S2**: UMR FL-3 swimming inside a 0.5-inch diameter tube at frequency of 10 Hz.
- **S3**: UMR FW-5 swimming inside a 0.5-inch diameter tube at frequency of 10 Hz.
- **S4**: UMR FW-2 swimming inside a 0.5-inch diameter tube at frequency of 10 Hz.
- **S5**: UMR FL-9 swimming inside a 0.375-inch diameter tube at frequency of 10 Hz.
- **S6**: UMR FL-3 swimming inside a 0.375-inch diameter tube at frequency of 10 Hz.
- **S7**: UMR FW-5 swimming inside a 0.375-inch diameter tube at frequency of 10 Hz.
- **S8**: UMR FW-2 swimming inside a 0.375-inch diameter tube at frequency of 10 Hz.
- **S9**: UMR FL-9 swimming inside a 0.25-inch diameter tube at frequency of 10 Hz.
- **S10**: UMR FL-3 swimming inside a 0.25-inch diameter tube at frequency of 10 Hz.
- **S11**: UMR FW-5 swimming inside a 0.25-inch diameter tube at frequency of 10 Hz.
- **S12**: UMR FW-2 swimming inside a 0.25-inch diameter tube at frequency of 10 Hz.
- **S13**: UMR FL-9 swimming inside a 0.1875-inch diameter tube at frequency of 10 Hz.
- **S14**: UMR FL-3 swimming inside a 0.1875-inch diameter tube at frequency of 10 Hz.
- **S15**: UMR FW-5 swimming inside a 0.1875-inch diameter tube at frequency of 10 Hz.
- **S16**: UMR FW-2 swimming inside a 0.1875-inch diameter tube at frequency of 10 Hz.
- **S17**: UMR FL-9 swimming inside an *ex vivo* aorta and iliac artery model at frequency of 10 Hz.
- **S18**: UMR FL-3 swimming inside an *ex vivo* aorta and iliac artery model at frequency of 10 Hz.
- **S19**: UMR FW-5 swimming inside an *ex vivo* aorta and iliac artery model at frequency of 10 Hz.
- **S20**: UMR FW-2 swimming inside an *ex vivo* aorta and iliac artery model at frequency of 10 Hz.

## Supplementary Information

Below is the link to the electronic supplementary material.

Supplementary file 1 (mp4 7712 KB)

Supplementary file 2 (mp4 4131 KB)

Supplementary file 3 (mp4 7578 KB)

Supplementary file 4 (mp4 17780 KB)

Supplementary file 5 (mp4 12210 KB)

Supplementary file 6 (mp4 10100 KB)

Supplementary file 7 (mp4 11363 KB)

Supplementary file 8 (mp4 16277 KB)

Supplementary file 9 (mp4 8103 KB)

Supplementary file 10 (mp4 5012 KB)

Supplementary file 11 (mp4 11535 KB)

Supplementary file 12 (mp4 11524 KB)

Supplementary file 13 (mp4 5439 KB)

Supplementary file 14 (mp4 3226 KB)

Supplementary file 15 (mp4 13346 KB)

Supplementary file 16 (mp4 6758 KB)

Supplementary file 17 (wmv 3138 KB)

Supplementary file 18 (wmv 4286 KB)

Supplementary file 19 (wmv 2161 KB)

Supplementary file 20 (wmv 4497 KB)

## Data Availability

The datasets generated and analyzed during the current study are available from the corresponding author on reasonable request.

## Declarations

### Materials availability

All materials used in this study are available from the corresponding author upon reasonable request.

### Code availability

The code used for data analysis, simulation, and visualization in this study is available from the corresponding author upon reasonable request.
